# Sugiol Masters Apoptotic Precision to Halt Gastric Cancer Cell Proliferation

**DOI:** 10.3390/ph16111528

**Published:** 2023-10-27

**Authors:** Tahani Bakhsh, Samah Sulaiman Abuzahrah, Safa H. Qahl, Mohamed A. Akela, Irfan A. Rather

**Affiliations:** 1 Department of Biological Science, College of Science, University of Jeddah, Jeddah 21589, Saudi Arabia; 2 Department of Biology, College of Sciences and Humanities in Al-Kharj, Prince Sattam bin Abdulaziz University, Al-Kharj 11942, Saudi Arabia; 3 Department of Biological Sciences, Faculty of Science, King Abdulaziz University, Jedddah 21589, Saudi Arabia

**Keywords:** human gastric cancer, sugiol, caspase cascade, inflammation, oxidative stress, cell-cycle arrest

## Abstract

Sugiol, a natural compound with anticancer properties, has shown promise in various cancer types, but its potential in preventing gastric cancer remains uncertain. In this study, we aimed to examine the inhibitory effect of sugiol on human gastric cancer cell proliferation. Our findings demonstrate that sugiol effectively suppresses the proliferation of SNU-5 human gastric cancer cells, leading to apoptotic cell death. We assessed the chemo-preventive potential of sugiol via an MTT assay and confirmed the induction of oxidative stress using the H2DCFDA fluorescent dye. Treatment with sugiol at concentrations higher than 25 µM for 24 h resulted in an increase in intracellular levels of reactive oxygen species (ROS). This elevation of ROS levels inhibited cell-cycle progression and induced cell-cycle arrest at the G1 phase. Furthermore, our study revealed that sugiol reduces the viability and proliferation of SNU-5 cells in a dose-dependent manner. Importantly, ADME and toxicity analyses revealed that sugiol was effective and nontoxic at low doses. In parallel, we utilized the Swiss target prediction tool to identify potential targets for sugiol. Enzymes and nuclear receptors were identified as major targets. To gain insights into the molecular interactions, we performed structure-based molecular docking studies, focusing on the interaction between sugiol and STAT3. The docking results revealed strong binding interactions within the active site pocket of STAT3, with a binding affinity of −12.169 kcal/mole. Sugiol’s -OH group, carbonyl group, and phenyl ring demonstrated hydrogen-bonding interactions with specific residues of the target protein, along with Vander Waals and hydrophobic interactions. These data suggest that sugiol has the potential to inhibit the phosphorylation of STAT3, which is known to play a crucial role in promoting the growth and survival of cancer cells. Targeting the dysregulated STAT3 signaling pathway holds promise as a therapeutic strategy for various human tumors. In combination with interventions that regulate cell cycle progression and mitigate the DNA damage response, the efficacy of these therapeutic approaches can be further enhanced. The findings from our study highlight the antiproliferative and apoptotic potential of sugiol against human gastric cancer cells (SNU-5). Moreover, the result underpins that sugiol’s interactions with STAT3 may contribute to its inhibitory effects on cancer cell growth and proliferation. Further research is warranted to explore the full potential of sugiol as a therapeutic agent and its potential application in treating gastric cancer and other malignancies characterized by dysregulated STAT3 activity.

## 1. Introduction

Gastric cancer is a complex and multifactorial disease, and its development is influenced by a variety of risk factors. It is one of the most common cancers and has a high mortality rate, particularly when it is diagnosed at an advanced stage [1,2]. According to recent research, these risk factors include chronic gastritis, *Helicobacter pylori* infection, pernicious anemia, gastric polyps, and a family history of gastric cancer [3,4]. In addition, lifestyle factors such as smoking and a diet high in salted and smoked foods have also been shown to increase the risk of developing gastric cancer. It is important to note that the development of gastric cancer is a complex process that involves the interaction between multiple risk factors, and further research is needed to fully understand the mechanisms behind this disease. Nonetheless, identifying and addressing these risk factors is crucial for preventing and managing gastric cancer.

Although *Helicobacter pylori*-mediated infections are one of the leading and primary causes of cancer, gastric cancer involves multiple causal factors [5]. In the year 1994, *H. pylori*-mediated infections were known to be classified as human class-I carcinogens associated with gastric cancer, as also confirmed by the International Agency for Research on Cancer (IARC) [6]. Researchers in the field identified that asymptomatic or non-specific symptomatic dyspepsia was associated with the early occurrence of gastric cancer. However, complications in advanced gastric cancer could be associated with body weight loss, anorexia, and persistent abdominal pain [4]. Persistent patterns of vomiting may also be an indication of pyloric stenosis. A lack of distinct symptoms leads to a delay in the diagnostic process [5]. Therefore, it has been reported that a higher percentage (80%) of such cancer patients is recognized during the advanced cancer stages, especially in the areas where early diagnostic tools and programs are unavailable [7]. Although standard curative therapy, also known as the surgical resection approach, is available to treat gastric cancer, gastric cancer tumors are usually diagnosed during the late advanced stage, resulting in no implementation of surgical methods [6]. Moreover, chemotherapy and radiotherapy as viable alternatives have shown less potential in curing gastric cancer with intermediate results [8]. Therefore, there is an urgency to identify a potential therapeutic strategy to suppress the rapid growth and development of human gastric cancer.

Compared to synthetic drugs, natural products have shown prolonged potential in treating different diseases, including a variety of cancers, with negligible adversary effects; thus, natural compounds play a critical role in improving and nurturing human life. It is predicted that drugs derived from natural resources will gain huge applications in treating various diseases, including cancers [7]. Recently, a comprehensive study has summarized the beneficial properties of a diterpenoid known as sugiol [9]. This study described that sugiol has antimicrobial activity, antioxidant capacity, anti-inflammatory actions, anticancer activity, antiviral potential, and cardiovascular properties. In this context, natural compounds have emerged as promising sources of new therapies with minimal side effects. Sugiol, a diterpenoid with a range of beneficial properties, has shown anticancer potential against several cancer types [10,11,12], including breast cancer, lung cancer, and prostate cancer. However, its effects on gastric cancer remain unexplored.

The signal transducer and activator of transcription 3 (STAT3) is a well-studied transcription factor that is known to regulate multiple signaling pathways [13]. STAT protein has seven different isoforms, including STAT 1, 2, 3, 4, 5A, 5B, and 6. This protein is known to be activated in the cytoplasm via Janus kinases (JAKs), which are composed of four different intracellular non-receptor tyrosine kinases, which further transduce cytokine-mediated signaling [14]. Among these various STAT isoforms, STAT3 is the most complex transcription regulator. STAT3 is critically involved in different biological functions—for example, proliferation and survival. It is known to be activated by IL-6, which is usually higher in the tumor microenvironment than that in benign tissues [15]. Studies demonstrated that gastric cancer patients have higher levels of IL-6 compared to non-cancerous patients. Hence, it is also proposed to be used as an indicator of gastric cancer diagnosis [16]. This study examines if sugiol can induce apoptosis in human gastric cancer cells and inhibit their proliferation by modulating STAT3. Additionally, sugiol’s ADMET properties indicate its efficacy and toxicity, which are easily predicted using computer-aided techniques.

The findings of this study could provide new insights into the development of natural compounds for the treatment of gastric cancer.

## 2. Results and Discussion

### 2.1. Sugiol Exhibits Anticancer Potential against Human Gastric Cancer Cells

**Figure 1:**
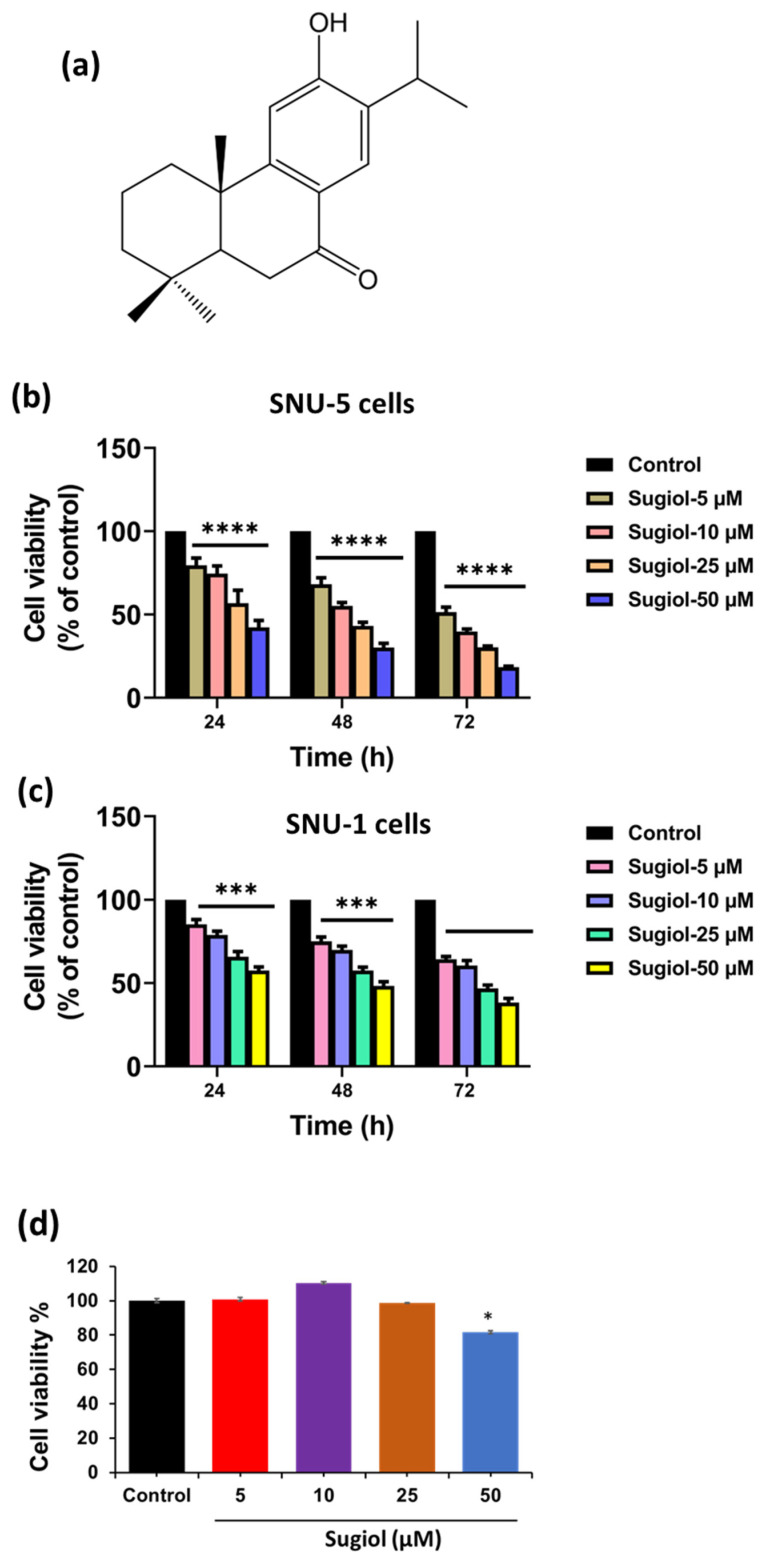
The effect of sugiol treatment against the human gastric cells. (**a**) The 2D chemical structure of sugiol. (**b**,**c**) The cell viability percentage of SNU-5 (**b**) and SNU-1 (**c**); human gastric cancer cells and (**d**) normal gastric cancer cells upon treatment with sugiol was determined using MTT assay in a dose-dependent manner. All experimental assays were conducted in triplicate, and the results are presented as the value of mean ± SE; the *p*-values are considered as * < 0.05, *** < 0.01, and **** < 0.001 compared to the control group.

Sugiol, a major component of *Metasequoia glyptostroboides* extract, has been found to significantly inhibit the growth of cervical cancer cells by activating the intrinsic apoptosis pathway [17]. Nevertheless, a lower cytotoxic effect of sugiol was observed towards normal cells than cancer cells, signifying that sugiol has more potential to target cancer cells than normal cells, as shown in Figure 1b,c [10]. To validate this observation, we also treated sugiol to normal, immortalized human gastric epithelial cells. As expected, similar results were also observed in our study. Sugiol was non-toxic at all the chosen concentrations except at 50 μM treatment (cell viability—81.6 ± 0.92) for 24 h. This particular concentration (50 μM) of sugiol significantly decreased (*p* < 0.05) the viability of normal human gastric epithelial cells when compared to non-treated control cells. Therefore, further, to evaluate the anticancer potential of sugiol, we treated sugiol with different doses (0–50 μM) for 24 h to SNU-5 human gastric cancer cells (Figure 1b,c). As observed, the sugiol treatment significantly suppressed the growth and proliferation of both cell lines in a dose-dependent manner. At 24 h, the concentration of sugiol treatment was increased, and it reduced the percentage of viable cells, 5 μM—79.53 ± 4.36 (*p* < 0.001), 10 μM—74.57 ± 4.66 (*p* < 0.001), 25 μM—56.83 ± 7.68 (*p* < 0.001), and 50 μM—42.27 ± 4.15 (*p* < 0.001), respectively. At 24 h, similar results were observed in the SNU-1 cell line after sugiol treatment (Figure 1c).

The results demonstrated in this study identified 34.89, 15.00, and 5.84 μM concentrations as an IC_50_ value of sugiol at different time points (24, 48, and 72 h), which remarkably suppressed the growth of SNU-5 cells (Figure 1b), respectively. Also, we observed 77.26, 44.70, and 19.66 μM the IC_50_ concentrations of sugiol in the SNU-1 cell line at different time points (24, 48, and 72 h), respectively. These findings are consistent with those of a study by Wang et al. [10], which demonstrated that sugiol significantly inhibited the growth of SKOV3 cells (IC_50_; 25 μM). Collectively, these results suggest that sugiol treatment has the potential to control the growth and proliferation of human gastric cancer cells without being cytotoxic to normal gastric epithelial cells at a particular concentration.

### 2.2. Morphological Analysis—Effect of Sugiol on SNU-5 Cells via SEM

**Figure 2:**
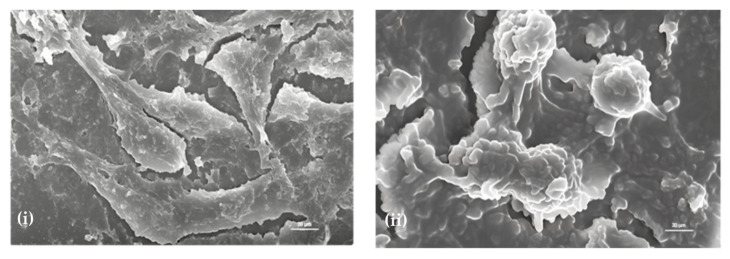
Determination of the morphological and internal cellular damage to SNU-5 cancer cells treated with sugiol via scanning electron microscopy (SEM) of (**i**) control and (**ii**) 25 μM sugiol-treated cells. Scale bar: 20 μm.

As depicted in Figure 2, distorted cell morphology of SNU-5 cells was observed upon sugiol treatment at the concentration of 25 μM when compared to control intact cells. A clear visual of distorted cell morphology supports the results of cell viability, i.e., at higher concentrations of sugiol. However, a slight modification of SNU-5 cell morphology was also observed even at the lower concentrations of sugiol treatment, i.e., 5 and 10 μM, when compared to control cells.

Interestingly, similar morphological changes were observed in the treated group, including cell shrinkage, membrane blebbing, cell fragments, and loss of cell membrane integrity, sequentially leading to cell death [15,16]. These findings evidenced the potential of sugiol as a therapeutic candidate for cancer therapeutics. The dose-dependent effect of sugiol on cancer cell morphology also indicates the careful optimization of dosage required to achieve optimal therapeutic efficacy with a minimal toxic effect on normal cells.

### 2.3. Sugiol Treatment Increases the ROS Generation and Membrane Potential

The effect of sugiol on the redox status of intracellular ROS was determined to demonstrate the role of the action mechanism of sugiol against SNU-5 gastric cancer cells. Earlier reports have confirmed the role of increasing amounts of intracellular ROS production in the progression of apoptosis [18]. Therefore, in this study, a fluorescent staining method employing H_2_DCFDA was used to determine comparative variations of intracellular ROS production between the SNU-5 gastric cancer cells treated with sugiol and cells without sugiol treatment. To this aim, SNU-5 gastric cancer cells treated for 24 h with sugiol at different concentrations were stained with H_2_DCFDA to visualize the change in the levels of intracellular ROS.

**Figure 3:**
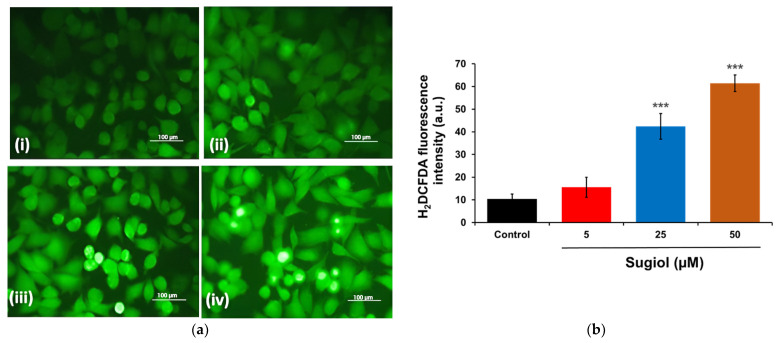
The effect of sugiol treatment on ROS generation was estimated. (**a**) The representative fluorescent images indicating the effect of sugiol treatment against the human gastric SNU-5 cancer cells. (i) Control; (ii) 5 μM; (iii) 25 μM; (iv) and 50 μM of sugiol. The scale bar of the images is 100 μm captured at 60× magnification. (**b**) The fluorescent intensity was represented, and the significance was estimated when compared to the control untreated group. All experimental assays were conducted in triplicate, and the results are presented as the value of mean ± SE; the *p*-values are considered as *** < 0.001 compared to the control group.

The results of fluorescent microscopy confirmed that the treatment of sugiol significantly increased the production of intracellular ROS in the sugiol-treated SNU-5 gastric cancer cells in a dose-dependent fashion (Figure 3a). Interestingly, SNU-5 gastric cells treated with sugiol displayed increasing fluorescence intensity compared to untreated control cells, indicating increased intracellular ROS production. Further results obtained from the quantitative measurement of intracellular ROS production confirmed that 50 µM of sugiol significantly (*p* < 0.001) increased the ROS level in SNU-5 cells by around 61.42 ± 3.66% compared to the control (Figure 3b). These results confirmed that the increased ROS level upon sugiol treatment induced cell death against the human gastric cancer cells. Similar results were also observed by Jung et al. [12], where they observed that sugiol treatment inhibited the activity of STAT3 via a mechanistic approach of transketolase inhibition, eventually leading to a drastic increase in the levels of ROS production and activation of MEG2, a classic tyrosine-specific protein tyrosine phosphatase, in DU145 prostate cells.

**Figure 4:**
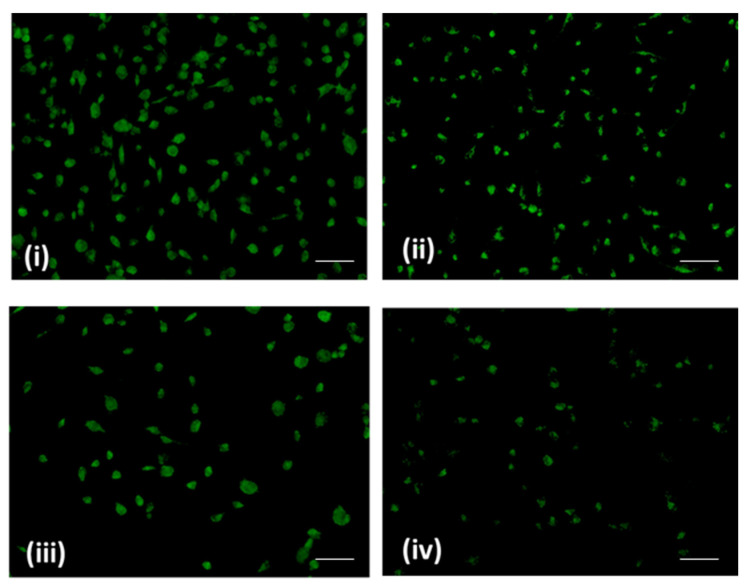
Mitochondrial membrane potential (∆ψm) via Rhodamine-123 staining detected by fluorescence microscopy. (**i**) Control and (**ii**) 5 μM (**iii**) 25 μM and (**iv**) and 50 μM of sugiol. Fluorescence images were captured at 20× magnification (scale bar = 0.1 mm).

Mitochondria are well known to play a critical role in mediating the intrinsic apoptosis pathway in mammalian cells [17,18]. Loss of mitochondrial membrane potential has been well identified as a hallmark of apoptosis induction. Rhodamine-123 dye is commonly used to examine the effect of treatments towards its effect on mitochondrial membrane potential [19]. Our results signify a loss of mitochondrial membrane potential (Δψm). Also, the fluorescence intensity of Rhodamine-123 in SNU-5 cells remarkably decreased in a dose-dependent manner (Figure 4). This revealed that sugiol possibly induced mitochondrial damage in SNU-5 cells upon treatment.

### 2.4. Sugiol Treatment Induced Cell-Cycle Arrest at G1 Phase

**Figure 5:**
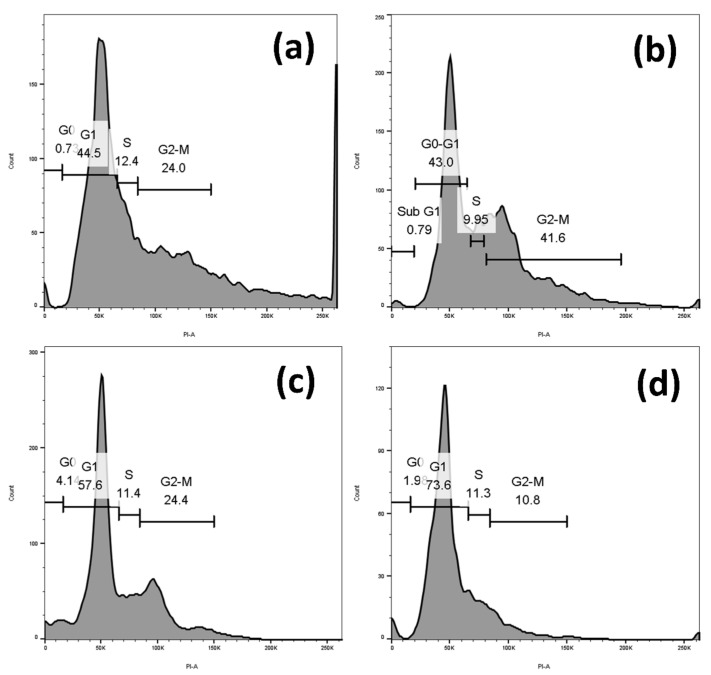
Effect of sugiol treatment against cell-cycle progression. The representative graph indicates the statistical significance when compared to the control group. All experimental assays were conducted in triplicate, and the results are presented as the value of mean ± SE; (**a**) Control; (**b**) 5 µM; (**c**) 25 µM; (**d**) 50 µM sugiol treatment.

A number of studies have highlighted that an increase in endogenous free radical generation contributes to cell cycle arrest as a consequence of the DNA damage response [19,20,21]. Therefore, we next investigated whether sugiol treatment-induced cell-cycle arrest in SNU-5 cells was due to the elevation of intercellular ROS. Since ROS are the main molecular drivers of several intrinsic pathways, to this aim, we examined their activity in sugiol-treated and untreated control SNU-5 gastric cancer cells. Our results displayed a significant elevation of the SNU-5 cells population at the G1 cell phase upon 24 h of sugiol treatment in a dose-dependent manner (Figure 5). Approximately 73.6% of the total cell population was arrested at the G1 phase of the cell cycle compared to untreated control cells, which confirmed the induction of cell-cycle arrest upon sugiol treatment against the human gastric cancer cells. The results of a similar pattern were also demonstrated by Fronza et al. [22], where sugiol influenced the cell cycle arrest and cell progression in different phases, primarily at the G1/G0 phase of the cell cycle.

### 2.5. Sugiol Altered the mRNA and Protein Levels of Apoptosis-Associated Genes in SNU-5 Cells

**Figure 6:**
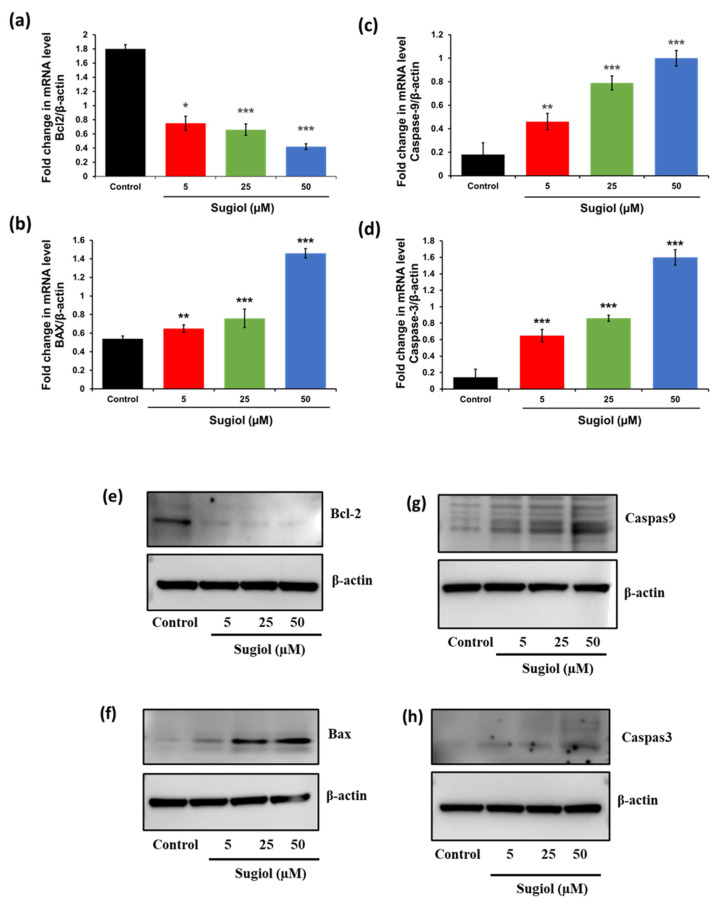
The effect of sugiol treatment was analyzed against the apoptosis signaling pathway. The representative graph indicates the statistical differences in terms of mRNA level between the treated and untreated groups against (**a**) Bcl2, (**b**) Caspase-9, (**c**) BAX, and (**d**) Caspase-3 genes. Represented protein expression of (**e**) Bcl-2, (**f**) Bax, (**g**) Caspase-9, and (**h**) Caspase-3 after treatment with or without sugiol at indicated concentrations. All experimental assays were conducted in triplicate, and the results are presented as the value of mean ± SE; the *p*-values are considered as * < 0.05, ** < 0.01, and *** < 0.001.

**Figure 7:**
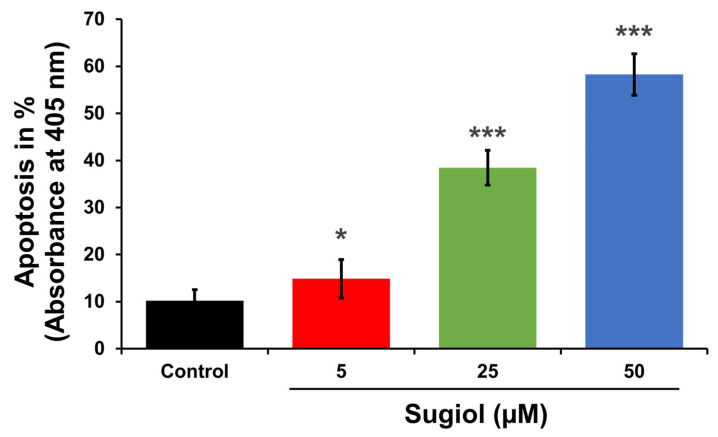
Sugiol treatment activated the apoptosis pathway. The representative graph indicates the statistical significance between the control and sugiol treated groups, indicating the increasing percentage of apoptotic cell population upon sugiol treatment in a dose-dependent manner. All experimental assays were conducted in triplicate, and the results are presented as the value of mean ± SE; the *p*-values are considered as * < 0.05, and *** < 0.001.

To confirm the activation of apoptosis, we performed quantitative PCR analysis and Western blot analysis to examine the mechanism underlying sugiol-induced apoptosis at the molecular level in SNU-5 gastric cancer cells. To this aim, we performed PCR analysis on a panel of hypoxia- and apoptosis-relevant genes. Here, we analyzed the transcriptional and translational expression of several important genes using the pooled control or sugiol-treated samples involved in the programmed cell death. The results revealed that sugiol treatment of SNU-5 cells showed a differential expression in the mRNA and protein levels of the key genes involved in the apoptosis pathway compared to untreated control cells, including Bcl-2, Bax, Caspase-9, and Caspase-3 (Figure 6). The relative gene expression was normalized by β-actin as a housekeeping gene using specific primers of these target genes. Our results showed consistency with our PCR array and Western blot data, along with a marked decrease in the transcriptional expression level of Bcl-2, an anti-apoptosis factor, in the sugiol-treated SNU-5 gastric cancer cells (Figure 6a,e). In contrast, the mRNA transcriptional and protein expression levels of pro-apoptosis genes, including BAX, Caspase-3, and Caspase-9, were significantly enhanced in sugiol-treated cells (Figure 6b–d,g,h). These results confirmed that sugiol treatment could activate the apoptosis signaling pathway against the human gastric cancer cells to suppress the growth and proliferation of cancer cells (Figure 7).

**Figure 8:**
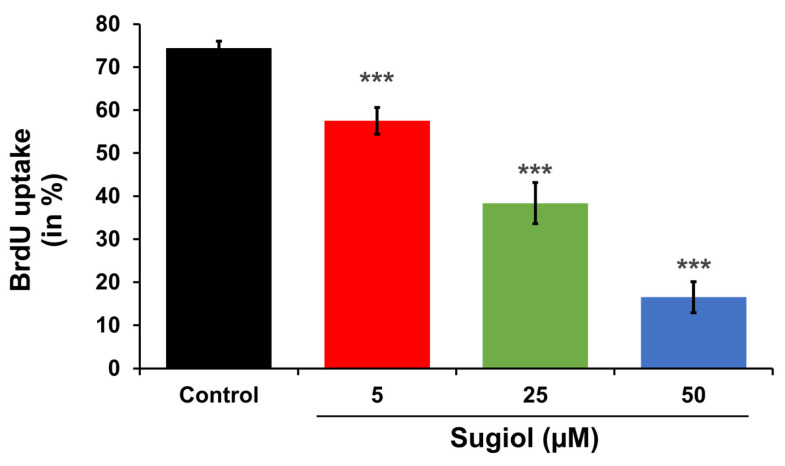
Effect of sugiol treatment on BrdU uptake. The representative graph indicates the statistical significance between the control and sugiol treated groups, indicating the decreased percentage of BrdU uptake upon sugiol treatment in a dose-dependent manner. All experimental assays were conducted in triplicate, and the results are presented as the value of mean ± SE; the *p*-values are considered as *** < 0.001.

In order to support all these observations, we performed BrdU cell uptake assays. As shown in Figure 8, the percentage of BrdU uptake by the cells significantly decreased in a dose-dependent manner.

### 2.6. Sugiol Inhibits STAT3 Signaling in SNU-5 Cells

**Figure 9:**
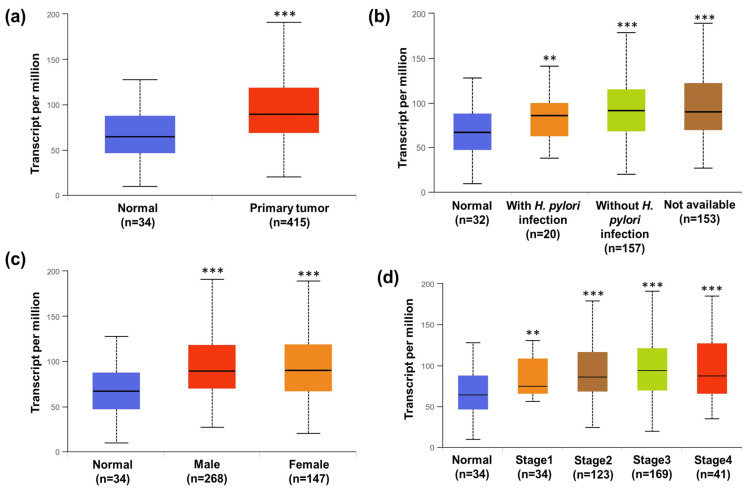
The STAT3 transcriptional level in stomach cancer patients. (**a**) The mRNA level of STAT3 was compared between normal and tumor patients. Significance was estimated with respect to normal, *** < 0.001. (**b**) The level of STAT3 was estimated in cancer patients with and without H. pylori infection. Significance was calculated with respect to normal (** < 0.01 and *** < 0.001). (**c**) The level of STAT3 transcriptional factor in male and female stomach cancer patients. Significance was estimated with respect to normal, *** < 0.001. (**d**) The transcriptional level of STAT3 was estimated in various stages of gastric cancer progression. The effect of sugiol treatment against the human gastric cells. Significance was estimated with respect to normal, ** < 0.01, *** < 0.001. The data were retrieved from ULCAN TCGA public database.

**Figure 10:**
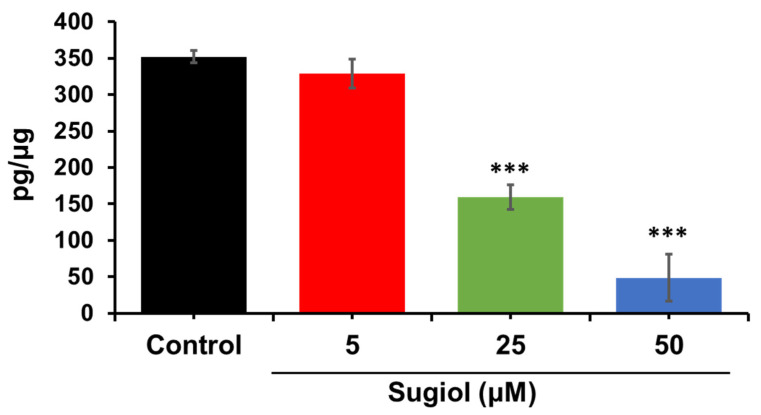
Effect of sugiol treatment on phospho-STAT3 (Tyr 705) in a dose-dependent manner. All experimental assays were conducted in triplicate, and the results are presented as the value of mean ± SE; the *p*-values are considered as *** < 0.001.

Numerous studies in the last few years have shown several pieces of evidence indicating the crucial role of aberrant STAT3 in malignant transformation and tumorigenesis [23]. As per the TCGA database, the STAT3 signaling pathway is significantly upregulated in malignant tumors in cancer patients when compared with non-malignant tissue of normal patients (Figure 9a). As we know, *H. pylori* infection is one of the factors that also contribute to cancer in the human gastrointestinal tract. Therefore, we also investigated the effect of *H. pylori* infection affecting STAT3 transcriptional levels in gastric cancer patients. As depicted in Figure 9b, tumors with *H. pylori* infection showed non-significant differences in the STAT3 level compared to tumor-bearing patients without *H. pylori* infection. This result indicates that *H. pylori* infection does not stimulate the STAT3 level, and STAT3 transcriptional upregulation was independent of *H. pylori* infection and was significantly higher compared to normal non-tumor-bearing patients. In addition, we also found upregulation of STAT3 transcription levels in gastric cancer patients irrespective of their gender (Figure 9c). STAT3 transcriptional levels in both male and female gastric cancer patients were significantly higher when compared to normal non-tumor-bearing patients. In fact, the data do not show any significance with regard to the STAT3 transcriptional level as cancer progression takes place from stages 1 to 4. All the stages have shown a significant upregulation in STAT3 level compared to normal non-tumor-bearing patients (Figure 9d). The results conclude that STAT3 is a crucial signaling molecule that is primarily activated in gastric cancer patients irrespective of *H. pylori* infection, gender, and stage of progression. Therefore, STAT3 could be a universal target for gastric cancer patients. Inhibition of STAT3 could be a potential target therapy for gastric cancer patients. Studies have confirmed that activated STATs form a dimer through phosphorylation at Tyr-SH2 domain interactions, and dimerization is essential for the DNA-binding activity of STATs [24]. Hence, disrupting or preventing the dimerization process can serve as an effective strategy to regulate the persistent activity of STAT3 and its associated functions [25]. This notion finds support in the observation that the mutation of the essential Tyr 705 residue in STAT3 to phenylalanine results in the inactivation of STAT3 signaling and the inhibition of its biological functions [12]. To investigate the efficacy of sugiol in inhibiting the phosphorylation of STAT3 at Tyr 705, we performed the ELISA assay upon 24 h of treatment with or without sugiol in a dose-dependent manner. As demonstrated in Figure 10, sugiol demonstrated its efficacy in suppressing the phosphorylation of STAT3.

### 2.7. In Silico ADMET Studies

The in silico ADMET parameters assessed the pharmacophore properties of sugiol using the SwissADME tool. Table 1 illustrates the results of the physicochemical properties of sugiol, considered a druggable compound, through the suitable molecular weight (MW), number of hydrogens, and rotatable bound, which presented the stability of the compound. The lipophilicity of sugiol is good and has a consensus log Po/w value of 4.64, indicating that it is lipophilic and can thus diffuse through the cell membrane. Furthermore, sugiol was predicted to have moderately soluble levels of aqueous. The results of pharmacokinetics also revealed that the sugiol has a high gastrointestinal (GI) absorption level with good Blood–Brain Barrier (BBB) penetration levels. Accordingly, this compound was anticipated to be safe against the central nervous system (CNS) because it cannot bind to the P-gp substrate. Moreover, the sugiol was predicted as a non-inhibitor of CYP1A2, CYP2D6, and CYP3A4 except CYP2C19 and CYP2C9. Also, the drug-likeness properties of the sugiol demonstrated that this compound is suitable for oral bioavailability. As defined by the LR5 rule, which evaluates the oral bioavailability of compounds based on their drug-like properties, this molecule was determined to have good drug-like features. In the context of medicinal chemistry, the structural analysis of this compound reveals it to be sugiol, confirming its compatibility with other compounds and suggesting that it can serve as a promising lead compound with the potential for enhancement through modifications. One noteworthy advantage of sugiol is its straightforward chemical synthesis, which simplifies the process of producing this compound. This accessibility can contribute to its feasibility as a drug candidate. Moreover, when considering the comprehensive set of properties, sugiol emerges as a compelling prospect for drug development. Furthermore, the Swiss ADME (Absorption, Distribution, Metabolism, and Excretion) profiles for sugiol, depicted in Table 1, offer a comprehensive overview of its pharmacokinetic characteristics and provide further evidence of its potential as an effective pharmaceutical agent.

#### 2.7.1. Toxicity Analysis

ProTox-II software was used to assess the toxicity of the sugiol. The server can be accessed through a web link https://tox-new.charite.de/protox_II/ (accessed on 23 October 2023). The toxicity classes were defined according to the globally harmonized system of classification, labeling, and chemicals. The results showed that the sugiol belonged to class 4, and the lethal dose (LD_50_) was 570 mg/kg. This finding confirms that the sugiol is non-toxic or low-toxic according to class 4, and sugiol at high doses will be harmful if swallowed (300 < LD_50_ ≤ 2000). Moreover, ProTox-II software assessed several toxicological parameters of sugiol, including hepatotoxicity, cytotoxicity, carcinogenicity, mutagenicity, immunotoxicity, nuclear receptor signaling pathways, and stress response pathways (Table 2). All sugiol targets were predicted to be inactive with high probability except mitochondrial membrane potential (MMP), which was predicted to be active with 60% of probability. These indications can be beneficial for utilizing sugiol as a less-toxic drug.

#### 2.7.2. Prediction of Targets and Docking Studies

**Figure 11:**
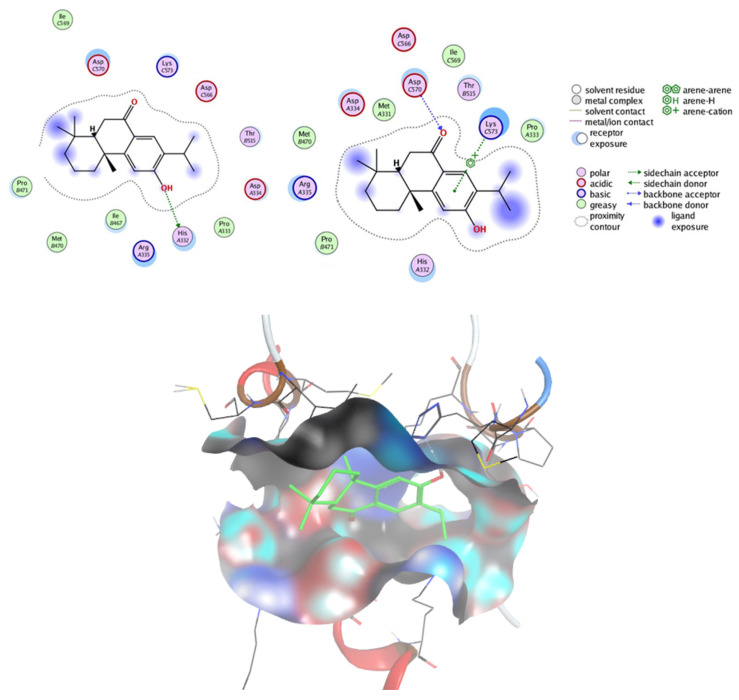
Molecular docking between sugiol and STAT3. The images show different 2D and 3D docking poses of the sugiol inside the active site pocket of the target.

The Swiss target prediction tool predicted enzymes and nuclear receptors as the major targets for sugiol. Since our studies established that sugiol has the potential to induce cell-cycle arrest at the G1 phase of SNU-5 gastric cancer cells, alter the mRNA level of apoptosis-associated genes, and inhibit STAT3 signaling, we performed structure-based molecular docking studies to illustrate the potential interactions between sugiol and STAT3. This approach allowed us to elucidate the potential binding interactions between sugiol and the STAT3 protein, shedding light on the molecular basis of its inhibitory effects. The strongest binding interactions of sugiol within the active site pocket are shown in Figure 11 with the binding affinity of −12.169 kcal/mole. The pocket was composed of Met331, His332, Pro333, Asp334, Arg335, Ile467, Met470, Pro471, Thr515, Asp566, Ile569, Asp570, Lys573. Interestingly, sugiol showed different interactions with STAT3 within this binding pocket. Notably, the -OH group, the carbonyl group, and the phenyl ring of sugiol were found to engage in specific hydrogen bonding interactions with key amino acid residues. The -OH group forms a crucial hydrogen bond with His332, the carbonyl group establishes a similar interaction with Asp570, and the phenyl ring engages in a hydrogen bond with Lys573. These interactions, in addition to Vander Waals and hydrophobic forces, collectively contribute to the robust binding observed between sugiol and STAT3. Our comprehensive molecular docking analysis provides compelling evidence that sugiol possesses the capacity to inhibit STAT3 phosphorylation. This inhibition holds significant implications for impeding the growth and proliferation of cancer cells. Our findings not only enhance our understanding of sugiol’s mechanisms of action but also highlight its potential as a promising candidate for the development of novel cancer therapeutics.

Persistent activation of STAT3, a member of the STAT family, is frequently observed in various types of human malignancies. This persistent activation of STAT3 is known to play a crucial role in promoting the growth and survival of cancer cells [26,27]. Essentially, STAT3 is a transcription factor that regulates the expression of genes involved in cell proliferation, survival, and immune responses. When STAT3 is aberrantly activated, it leads to the dysregulation of these genes, contributing to tumor development and progression.

To combat the detrimental effects of STAT3 activation in cancer, researchers have explored different strategies. One approach involves using antisense RNA or small interfering RNA (siRNA) to specifically target and reduce the levels of STAT3 in cancer cells. By doing so, they have successfully triggered apoptosis, or programmed cell death, in cancer cells and induced tumor regression [28,29]. This highlights the critical role of STAT3 in maintaining the survival of cancer cells and suggests that inhibiting its activity could be a promising therapeutic strategy. Several such inhibitors have been developed, and they hold promise in targeting various types of cancer. Examples of small molecule STAT3 inhibitors for cancer treatment include Stattic, WP1066, S3I-201, C188-9, SH-4-54, Napabucasin (BBI608), and TTI-101. These inhibitors work by disrupting the activation, dimerization, phosphorylation, or nuclear translocation of STAT3, thereby inhibiting its transcriptional activity and downstream signaling pathways that promote cancer cell survival and growth. Clinical trials and preclinical studies continue to evaluate the efficacy and safety of these inhibitors, with the hope of providing more effective and targeted therapies for cancer patients in the future.

The dysregulated STAT3 signaling pathway has also been implicated in various types of human tumors, including but not limited to breast cancer, lung cancer, colorectal cancer, and prostate cancer. Therefore, targeting this aberrant STAT3 activity holds great potential as a universal therapeutic strategy for treating a wide variety of human malignancies [26,27]. By specifically blocking STAT3 signaling, it may be possible to disrupt the growth-promoting and survival mechanisms employed by cancer cells across different tumor types. In addition to STAT3, other factors contribute to the development and progression of cancer. Transitions between different phases of the cell cycle, which are tightly regulated, are dependent on phosphorylation events. Furthermore, an increase in the generation of endogenous free radicals can lead to DNA damage and activate a DNA damage response, which can result in cell-cycle arrest. Therefore, these findings suggest that apart from targeting STAT3, interventions that regulate cell-cycle progression and mitigate DNA damage response may also be beneficial in treating cancers characterized by dysregulated STAT3 activity.

Overall, the persistent activation of STAT3 in various human malignancies is a critical driver of tumor growth and survival. Targeting this dysregulated STAT3 signaling pathway through different therapeutic approaches, including the use of small molecule inhibitors, holds promise for the effective treatment of a wide range of human tumors with abnormal STAT3 activity. Further research is needed to explore the full potential of these therapeutic strategies and their combination with other interventions to enhance their efficacy in combating cancer.

## 3. Materials and Methods

### 3.1. Chemicals and Reagents

Sugiol (PubChem CID: 94162) was purchased from Sigma Chemical Co. (St. Louis, MO, USA). Fetal bovine serum was purchased from Gibco (Waltham, MA, USA). RPMI-1640 medium, trypsin, 3-(4,5-dimethylthiazol-2-yl)-2,5-diphenyltetrazolium bromide (MTT), dimethyl sulfoxide (DMSO), and penicillin–streptomycin antibiotic cocktail was obtained from Sigma-Aldrich (St. Louis, MO, USA). Antibodies such as Bcl2 (2876), Bax (2772), Caspase-9 (9508), Caspase-3 (9662), and β-actin (4967) were purchased from Cell Signaling (Danvers, MA, USA). Other reagents and chemicals used in the experiments were of superior quality. Sugiol’s chemical structure is shown in Figure 1a.

### 3.2. Cell Culture and Maintenance

The human gastric cancer cells (SNU-5 and SNU-1 cell lines) were purchased from ATCC culture collection and grown in RPMI-1640 medium, whereas the normal yet immortalized human gastric epithelial cells (#ABC-HP021X cell line) were procured from AcceGen (Fairfield, NJ, USA). The cell lines were supplemented with 10% heat-inactivated fetal bovine serum (Gibco, Waltham, MA, USA), and the growth of these cell lines was maintained in 5% CO_2_ incubator at 37 °C.

### 3.3. Cell Viability Analysis

To evaluate the anticancer potential of sugiol, we treated sugiol on SNU-1 and SNU-5 cells in a time- (24, 48, and 72 h) and concentration-dependent (5, 10, 25, 50 µM) fashion. The cells were seeded in 96-well plates at a density of 2 × 10^4^ and allowed to grow until 80% confluence was reached. To assess sugiol’s anticancer potential against gastric cancer cells (SNU-1 and SNU-5), cells were treated with different concentrations of sugiol (5, 10, 25, and 50 μM). A fresh RPMI-1640 culture medium, containing 10 mL of MTT (5 mg/mL), was added to each well after 24 h of incubation, followed by a further 4 h incubation at 37 °C. After that, the culture medium from the wells was again removed, the insoluble crystals of formazan formed were dissolved in 50 μL DMSO, and the plates were incubated for 30 min in an ELISA plate shaker (LabTech, Sorisole, Italy). A TECAN microplate reader (200 Infinite PRO) was set at 540 nm to measure the absorbance, as previously mentioned [21]. The percentage of live and dead cells was calculated using the following formula: (treatment cell absorbance/control cell absorbance) × 100.

### 3.4. Confirmation of Cell Death via SEM Morphology Analysis

Techniques of SEM and bio-TEM were employed to observe the morphological changes in SNU-5 cells induced by sugiol. For SEM analysis, SNU-5 cells were propagated on activated glass chips, followed by washing with 1x-PBS. Cells were then fixed with glutaraldehyde (2.5%) and dehydrated using gradient exposure of ethanol. Next, drying of the glass chips was performed on a desiccator, deposited on SEM platform, super-coated with gold, and visualized under the SEM analyzer. After transfection with sugiol, the SNU-5 cells treated with sugiol were collected to observe the apoptotic morphology of the cells, followed by their fixation. Afterward, the cells were frozen and sectioned. Sections of the cells were placed on a copper grid and viewed under a transmission electron microscope.

### 3.5. H_2_DCHFDA Staining

For the ROS staining analysis, we treated the SNU-5 with 0, 5, 25, and 50 μM of sugiol for 24 h. Following treatment, cells were processed according to published protocols for fluorescence analysis [17]. Briefly, after 24 h of sugiol treatment, cells were washed using phosphate-buffered saline (PBS) and fixed using ice-cold 4% paraformaldehyde for next 10 min. The fixed cells were washed with PBS and incubated with 20 μM 2′,7′-dichlorodihydrofluorescein diacetate (H_2_DCFDA) solution for 30 min at 37 °C. Cells were re-washed with PBS and imaged using a fluorescence microscope. The total ROS fluorescence (Mean Green Area) was measured using ImageJ software version 1.53t via plugin for RGB measurements.

### 3.6. Mitochondrial Membrane Potential (∆ψm)

The effect of sugiol on mitochondrial membrane potential was evaluated in 24-well plates treated with or without 0, 5, 25, or 50 μM of sugiol. An inverted microscope was used for image analysis after washing the cells in PBS and staining them with Rhodamine-123 (1 g/mL) [30].

### 3.7. Cell-Cycle Profiling

As previously mentioned by Dey and Kang [21], the cell-cycle profiling was measured with some minor modifications. Briefly, SNU-5 gastric cancer cells were seeded at a density of 2.5 × 10^5^ cells/well in a 6-well plate and cultured in RPMI-1640 medium, followed by treatment with sugiol at different concentrations for 24 h. Treated cells were then harvested using 0.05% trypsin-EDTA, followed by gentle washing with 1x PBS. After washing, cells were fixed using 70% ice-cold methanol. Finally, the cell pellet was re-suspended in 1x PBS (0.1 mL), and 100 mg/mL of RNase (2 mL) was added and further incubated for an hour at 37 °C. Cells were further stained with propidium iodide (PI) solution (50 μg/mL) and vortexed smoothly. Cells were incubated in the dark for next 15 min. In each tube containing cell suspension, 1x ice-cold PBS (400 mL) was then added and followed for further analysis using flow cytometric analysis using 15 mW 488 nm laser. Cell Quest Pro-Software version 5.1 xvn was used to analyze the data.

### 3.8. Quantitative PCR Analysis

To analyze the effect of sugiol on gene expression of human gastric cancer cells, we performed quantitative polymerase chain reaction (PCR). To this aim, we extracted total RNA from SNU-5 cells using Tri-reagent, as reported by Dey and Kang [31]. Total RNA (2 mg) was treated with 2 units of DNAse (Invitrogen, Waltham, MA, USA) to perform reverse-transcription PCR using commercial iScript cDNA synthesis kit (Bio-Rad, Hercules, CA, USA). The Go Taq qPCR Mastermix was procured from Promina, New York, NY, USA, to perform q-PCR using a Real-Time PCR thermocycler (#CFX96; Bio-Rad). To compare the effect of sugiol with vs. without treatment on mRNA level, fold-change expression was measured and normalized using a housekeeping gene (i.e., β-actin), and ^ΔΔ^Ct method was used for analysis.

### 3.9. Analysis of Bromodeoxyuridine (BrdU) Uptake

For BrdU-uptake analysis, colorimetric test (#11647229001, Merck, Rahway, NJ, USA) was performed as per kit’s instruction. After treatment with different concentrations of sugiol, absorbance of reaction mixture was recorded at 450/690 nm.

### 3.10. Immunoblotting

After the indicated time point, cells were collected and lysed, and the supernatants (proteins) were saved for immunoblot analysis. SDS-PAGE was used to separate proteins and later transfer them onto nitrocellulose membranes (Millipore, Bedford, MA, USA). Membranes were rinsed twice with Tris-buffered saline (TBS-T) containing 0.05% (*v*/*v*) Tween 20 and incubated with 5% (*w*/*v*) non-fat dried milk prepared in TBS-T. After overnight incubation with primary antibody, membranes were washed and incubated with secondary antibodies conjugated to horseradish peroxidase for 2 h. Later, enhanced chemiluminescence (ECL) was used to visualize protein bands.

### 3.11. Apoptosis Assay

To evaluate the effect of sugiol on apoptosis, we used the previously described methodology [21]. Apoptosis induction was achieved with or without varying concentrations of the drug dose in SNU-5 cells.

### 3.12. STATs Transcriptional Level in Human Gastric Cancer Patients

To analyze the STATs transcriptional level in human gastric patients, we retrieved the patient data from The Cancer Genome Atlas (TCGA) pipeline option of ULCAN web source, as reported by Vadlamudi et al. [23]. The statistic was created with respect to the control normal group (*n* = 34).

### 3.13. ELISA Assay

To estimate the level of phosphorylated form of STAT3 in gastric cancer cells with and without treatment, we performed the ELISA assay. To this aim, cells were cultured with or without sugiol treatment in a dose-dependent manner. Further, cell lysate was collected, and we performed the ELISA as per the protocol instructed by the manufacturer (#DYC4607B, R&D Systems, Minneapolis, MN, USA). Finally, the absorbance was recorded using an ELISA reader.

### 3.14. Docking Studies

The crystallographic data for the STAT3 protein (PDB ID: 6QHD) with a resolution of 2.85 Å were sourced from the Protein Data Bank. To prepare the protein for analysis, we eliminated all heteroatoms, which encompassed the double-stranded DNA and crystallographic water molecules. We retained chain A as a STAT3 monomer for our investigations. The native pTyr peptide found in the 6QHD crystal structure was deliberately excluded to explore the competitive binding of sugiol within the pTyr705 peptide pocket. To optimize the protein structures, we conducted energy minimization using the Amber10 force field. The target protein’s binding pocket was defined using predefined MOE settings. The molecular structure of sugiol was constructed using ChemBioDraw Ultra 14.0 and subsequently saved in SDF format. These files were then imported into MOE, where the 3D structures were protonated and subjected to energy minimization using the MMFF94x force field. For the docking analysis, we employed the Molecular Operating Environment (MOE) software. To validate the target receptor, we performed docking simulations with the co-crystallized ligand and calculated the RMSD values to compare the docked conformations with the crystal structure. Subsequently, we further scrutinized and visualized the results generated by MOE software version (MOE 2015.10) using both MOE and Discovery Studio 4.0 software tools.

### 3.15. Statistical Analysis

The study’s findings were evaluated and expressed as the mean ± standard deviations (SDs). To assess the statistical distinctions among the groups, we conducted a one-way analysis of variance (ANOVA), followed by Tukey’s post hoc comparison test. Additionally, for comparisons involving multiple groups, we utilized Dunnett’s post hoc comparison test. Statistical significance was denoted as follows: * for *p*-values < 0.05, ** for *p*-values < 0.01, and *** for *p*-values < 0.001.

## 4. Conclusions

This study demonstrates that sugiol could arrest the human gastric cancer cell cycle at the G1 phase, effectively suppressing the growth of cancer cells. Sugiol’s ROS-mediated pathway induced the DNA damage response and increased the population of cells at the G1 phase, highlighting its chemo-preventive potential and potential application in drug development. The findings of this study provide new insights to further investigate the precise mechanism of action of sugiol at the molecular level, leading to permanent cell-cycle arrest against gastric cancer cells. Moreover, given that gastric cancer is a major global health concern, understanding the underlying mechanisms of its development and progression is critical for developing effective treatments. Our results also suggest that sugiol may be a promising alternative to conventional chemotherapy due to its lower cytotoxicity towards normal cells, thus potentially reducing unwanted side effects. Further studies are warranted to investigate the therapeutic potential of sugiol in preclinical and clinical settings and to develop novel therapeutic strategies for treating gastric cancer.

## Figures and Tables

**Table 1:** Identification of pharmacokinetic properties of sugiol, showing physicochemical properties, lipophilicity, water solubility, drug likeness, and medicinal chemistry.

Properties	Parameters	
Physicochemical Properties	Formula	C_20_H_28_O_2_
	Molecular weight	300.44 g/mol
	Num. heavy atoms	22
	Num. arom. heavy atoms	6
	Fraction Csp3	0.65
	Num. rotatable bonds	1
	Num. H-bond acceptors	2
	Num. H-bond donors	1
	Molar Refractivity	92.06
	TPSA	37.30 Å^2^
Lipophilicity	Consensus Log Po/w	4.64
Water Solubility	Log S (ESOL)	−5.38
Pharmacokinetics	GI absorption	High
	Log Kp (skin permeation)	−4.14 cm/s
Drug Likeness	Lipinski	Yes; 0 violation
	Bioavailability Score	0.55
Medicinal Chemistry	PAINS	0 alert
	Leadlikeness	No; 1 violation: XLOGP3 > 3.5
	Synthetic accessibility	3.45

**Table 2:** Toxicity profile analysis of sugiol using the toxicity model computation tool and online database.

Classification	Target	Prediction	Probability
Organ toxicity	Hepatotoxicity	Inactive	0.71
Toxicity end points	Carcinogenicity	Inactive	0.73
	Immunotoxicity	Inactive	0.88
	Mutagenicity	Inactive	0.92
	Cytotoxicity	Inactive	0.87
Tox21—nuclear receptor signaling pathways	Aryl Hydrocarbon Receptor (AhR)	Inactive	0.93
	Androgen Receptor (AR)	Inactive	0.77
	Androgen Receptor Ligand Binding Domain (AR-LBD)	Inactive	0.86
	Aromatase	Inactive	0.96
	Estrogen Receptor Alpha (ER)	Inactive	0.58
	Estrogen Receptor Ligand Binding Domain (ER-LBD)	Inactive	0.60
	Peroxisome Proliferator Activated Receptor Gamma (PPAR-Gamma)	Inactive	0.99
Tox21—stress response pathways	Nuclear factor (erythroid-derived 2)-like 2/antioxidant responsive element (nrf2/ARE)	Inactive	0.94
	Heat shock factor response element (HSE)	Inactive	0.94
	Mitochondrial Membrane Potential (MMP)	Active	0.60
	Phosphoprotein (Tumor Suppressor) p53	Inactive	0.87
	ATPase family AAA domain-containing protein 5 (ATAD5)	Inactive	0.96

## Data Availability

Data is contained within the article.
